# Soluble nitrogen forms in sand soil of Pallag: a quantitative report

**DOI:** 10.12688/f1000research.25260.2

**Published:** 2020-10-07

**Authors:** Ida Kincses, Jesus R. Melendez, Lenin Ramírez-Cando, Diego Burbano-Salas, Daniel Lowy, Gerardo Cuenca Nevarez, Viviana Talledo Solórzano, Juan Morales Arteaga, Benito Mendoza, Zsolt Sándor

**Affiliations:** 1 Institute of Agrochemistry and Soil Science, University of Debrecen, Debrecen, Hungary; 2 Research Group of Applied Plant Glycobiology, Dama Research Center limited, Kowloon, Hong Kong; 3 Facultad Educación Técnica para el Desarrollo, Universidad Católica de Santiago de Guayaquil, Guayaquil, Ecuador; 4 School of Biological Sciences and Engineering, Yachay Tech University, Hda. San José s/n y Proyecto Yachay, Urcuquí, Ecuador; 5 Carrera de Ingenería Ambiental, Escuela Superior Politécnica de Chimborazo, Riobamba, Ecuador; 6 VALOR HUNGARIAE Ltd, Budapest, Hungary; 7 Facultad de Ciencias Zootécnicas, Universidad Técnica de Manabí, Avenida Jose Maria Urbina, Portoviejo, Ecuador; 8 Biotechnical Faculty, University of Ljubljana, Ljubjlana, Slovenia; 9 Universidad Nacional de Chimborazo, Riobamba, Ecuador

**Keywords:** pot experiment, total N, nitrate, nitrite, ammonium, nutrient supply capacity

## Abstract

Nitrogen (N) is a crop macronutrient of major importance, which affects both plant growth and yield. In this paper we discuss the humus content (%) and various soluble N forms (NO _3_^-^, total N, nitrate-N, ammonium-N, and organic nitrogen) available in humus sand soil samples originating from the Pallag Experimental Station of Horticulture at the University of Debrecen, Hungary. We found 45.4% nitrate-N and 13.8% nitrite-N of total N content present in the soil. Considering the percentage distribution of soluble N forms present at the Pallag Experimental Station, we recommend using this soil in further pot experiments, given that this has optimal nutrient supply capacity. In addition, we examined possible statistical correlations between humus% and N forms.

## Introduction

Nitrogen (N) is an essential element for plants, takes various forms in soil ^1^, and is one of the most limiting nutrients for various crops ^2– 4^. Nitrogen is present in soils as inorganic nitrogen (nitrate, ammonium, and dinitrogen) and organic nitrogen (urea and amino acids) ^5^.

Conversions between organic and mineral N forms are primarily affected by soil microorganisms, which enable conversion to forms that plants can uptake ^6^. In addition, some pollutant N forms (e.g., nitrite) are present in soil, so nitrogen conversions may indirectly affect human health and load the environment ^1^. For this reason, quantifying studies aim to better understand nitrogen form ratios in soil and contribute to reaching sustainable agriculture practice. Recently, Jakab published a similar study ^7^ quantifying another vital element for plants, phosphorus, in soil from the same region.

Humus is a main fertility component of the soil, 65–75% of its structure being made up of organic matter ^8, 9^. We decided to include humus content in this N-related study as it represents a known indicator of soil quality ^10^.

Calcium chloride (CaCl _2_)-soluble nitrogen forms are the most available N ions for assimilation by plants ^11, 12^. Therefore, we quantified all N forms in CaCl _2_ solution. Additionally, we determined nitrate-N via a potassium chloride (KCl)-based method.

The main objectives of our study were: (a) to map out all easily absorbed nitrogen forms for better understanding of available nutrient composition and (b) to determine the ratio of pollutant (nitrite) and absorbable (ammonium ion, nitrate) N forms in humus sand soil in pot experiments.

## Methods

Randomized soil sampling, approx. 60 kg; 15 cores from control parcel (no fertilizer was applied, nor crop production, or land-use), was done at the Pallag Experimental Station of Horticulture at the University of Debrecen, Hungary on May 20, 2020, according to Hungarian national standard MSZ 080202 ^13^ from the -20 cm of topsoil using a vane. On other parcels (different from control parcels), orchards are being cultivated. Sampling point is located on a moderately hot and dry micro-region, the average annual temperature varies between 9.7-10.0 °C. The annual precipitation is of only 520–550 mm ^14^. Next, samples were transferred for measurements to a greenhouse (where pots had been placed for pot experiments); the greenhouse was located at Department of Agriculture, at the University of Debrecen. To measure chemical parameters, samples were sieved through a 2 mm mesh.

We determined soil moisture content gravimetrically, according to Klimes-Szmik ^15^, drying the soil samples at 105°C for 24 h and weighing the mass loss. To evaluate texture, Arany-type plasticity index was measured, using the methodology recommended by Hungarian national standard MSZ-08 0206/1-78 ^16^; briefly from a burette distilled water is added to 100g sample until soil reaches the upper limit of its water holding capacity. Soil pH was determined in 1 mol L ^-1^ KCl solution (soil/water = 1.0/2.5 wt./wt.), according to Buzás ^17^ using a glass electrode (Model Seven2Go Advanced Single-Channel Portable pH Meter, Mettler, Toledo. We first determined organic-C%. content with potassium dichromate, according to Székely ^18^, and than we calculated humus% from organic-C% according to: the Hungarian standard MSZ-08 0210-77 ^19^ (E2); briefly, 10 mL K-dichromate was added to 1.0 g of soil sample (solution/soil=10/1) in a 300 mL Erlenmeyer flask, then 0.10 g Ag _2_SO _4_ aqueous solution was added, boiled for 5 minutes, cooled to room temperature, and titrated with 0.2 N Mohr's salt solution. Ferroin was used was the indicator, given that its color change is reversible, pronounced, and fast. We calculated C% according to Equation (E1):

$$C\%=\frac{a*0.0006*100}{b}\;(\text{E}1)$$

where *a* is the volume difference (Mohr’s salt solution loss) between blank and soil sample titration (expressed in mL), multiplied by the Mohr-salt factor (which is 0.0006) and b represents the weight of the soil sample (g), multipled with 100 to convert results to percentage.

Next, humus% was calculated according to Equation (E2):

Humus(%)=C(%)∗1.724(E2)

Where C(%) is organic-Carbon (expressed in percentage), 1.724 is a multiplication factor which was determined by the Hungarian standard MSZ-08 0210-77 based on a number of experimental results; it was concluded that Humus(%) values can be calculated from organic-C% with high reliability.

All measurements of soluble N forms in soil were conducted according to Hungarian standard MSZ 20135:1999 ^20^; briefly, to determine KCl-NO _3_ (mg/kg) content, the sample was prepared with 1 mol L ^-1^ KCl of soil solution (74.5g KCl were added to 1 L distilled water ^21^. Spectra were recorded with a Model PU 8610 UV/VIS kinetics spectrophotometer by Pye Unicam (Cambridge, GB). For assessing total soluble nitrogen (UV degraded) content, soil samples were extracted with 0.01 mol L ^-1^ CaCl _2_ solution. Soil samples were mixed with sodium tetraborate buffer, then oxidized with excess K _2_S _2_O _8_ and passed into an ultraviolet digester. Nitrate was reduced on a cadmium-copper column and then converted to a colored azo compound via the Griess-Ilosvay reaction; dissolving 0.50 g of sulfanilic acid and 0.05 g of 1-naphthylamine in 150 mL of dilute acetic acid. A colored solution was obtained, which was analyzed by visible photometry at 540 nm (SKALAR photometry (San Plus Analyser, S.F.A.S).

We determined CaCl _2_-ammonium-N (mg/kg) using a modified Berthelot reaction-based method in which ammonia was initially converted to monochloramine and then to 5-aminosalicylate, according to Buzás ^17^ using 1% EDTA solution and 1 ml 0.06 N NaOH. After oxidation, a green color complex was obtained, with a maximum light absorption at 660 nm. Nitrate-N was determined as described above for the measurement of N content (after reduction, conversion to azo dye, and photometric measurement at 540 nm). All soluble forms were detected by SKALAR photometry (San Plus Analyser, S.F.A.S) in a segmented continuous flow (SCF) system. Finally, we calculated nitrite-N (mg/kg) using Equation (E3), proposed by Buzás ^17^:

OrganicN=TotalN−ammoniumN−(nitrateN+nitrite)(E3)

To better understand the correlation between humus% (Y) and N-forms (X), we carried out two different tests (i) the Pearson test to establish the relation between the variance (Y) and covariance (XY); and (ii) the Kendall test to verify whether the two variables may be regarded as statistically dependent. All statistical analysis was performed with R Statistical Software 3.5.1 (Foundation for Statistical Computing, Vienna, Austria).

## Results

**Figure 1.**
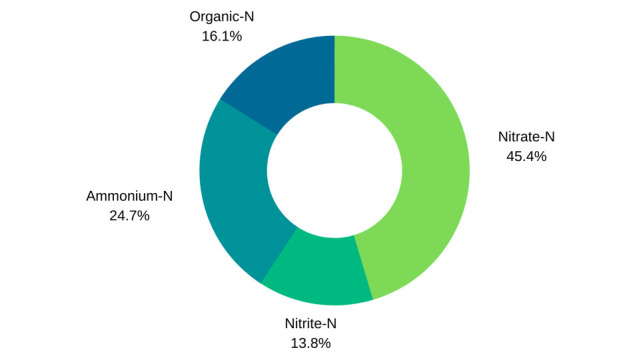
Percentage distribution of the soluble forms of nitrogen present in soil.

Soil pH was slightly acidic: pH (KCl) 5.5. As K _A_ = 30, according to Arany the soil is considered humus sand ^18^. For these samples, 1.4 % humus content was found, which represents a reasonable value for a sand soil ^18^. Nitrate content was determined using two methods: KCl-NO _3_ (12.66 mg/kg) and CaCl _2_-NO _3_ (13.53 mg/kg), and the average value of nitrate content (12.48 mg/kg) served for the calculation of percentages in Figure 1.

Average ammonium-N was 6.8 mg/kg, which represents 24.7% of the total-N content. In addition, 3.8 mg/kg of nitrite-N (pollutant nitrogen form) was assessed and found to be one order of magnitude less than the nitrate-N content, as expected for well-ventilated sand soil. Organic-N of 4.4 mg/kg contributed by 16.1% to the total nitrogen content of soil ( Table 1).

**Table 1.** Average values of humus%, and soluble N forms.

	Humus %	N forms (mg/kg)				
		Nitrate-N	Ammonium-N	Nitrite-N	Organic-N	Total-N
ID	1	2	3	4	5	6
avg Result	1.4	12.5	6.8	3.8	4.4	27.5
SD	0.15	0.075	0.02	derived parameter	0.15	0.05

**Figure 2.**
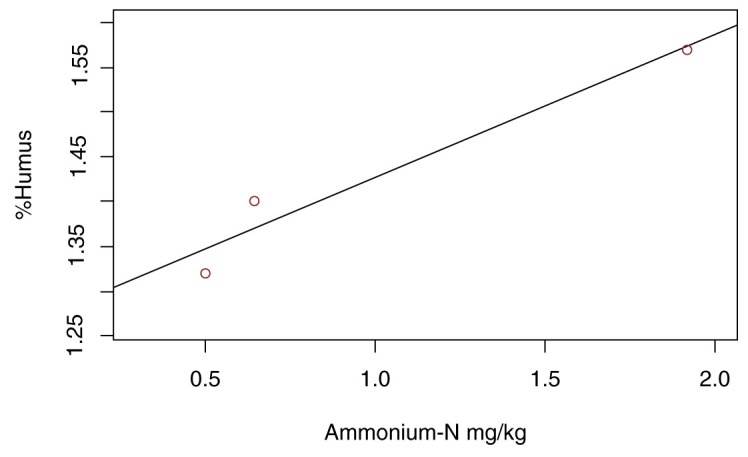
Correlation between humus% and ammonium-N (mg/kg).

Considering all forms of N under study, the results of correlation analysis evidenced that only the ammonium-N form was in strong correlation with humus%, with r ≈ 0.97 and T ≈ 0.99 ( Figure 2).

These promising initial results call for additional experiments to confirm the strong correlation between these two variables. Total-N also showed a good correlation with humus%; however, it was not as strong as that found between humus% and ammonium-N.

## Conclusions

Our results reveal that the ratio of nitrate-N and nitrite-N (which is determined mostly by soil oxidation-reduction conditions) is optimal in sand soil samples originating from the Pallag Experimental Station of Horticulture at the University of Debrecen, Hungary. Based on the percentage distribution of soluble N forms present at Pallag Experimental Station, authors recommend using this soil in further pot experiments, given the soil’s optimal nutrient supply capacity ^21^.

## Data availability

### Underlying data

Figshare: Supporting data - N forms and Humus% in Sand soil, Nyírség. https://doi.org/10.6084/m9.figshare.12581303.v3 ^22^

Data are available under the terms of the Creative Commons Attribution 4.0 International license (CC-BY 4.0).
