# Supplementary material for: Soluble nitrogen forms in sand soil of Pallag: a quantitative report
Source: F1000Res. 2020 Oct 7;9:781. Originally published 2020 Jul 28. [Version 2] doi: 10.12688/f1000research.25260.2 (PMC7542254; doi:10.12688/f1000research.25260.2)
Supplement: Peer review history

# Peer review: Reviewer response for version 2

**DOI:** 10.5256/f1000research.29978.r72580

**Published:** 2020-10-23

**Authors:** Yuhua Kong

**Affiliations:** 1 College of Forestry, Henan Agricultural University, Zhengzhou, China

Is the work clearly and accurately presented and does it cite the current literature?

Partly

If applicable, is the statistical analysis and its interpretation appropriate?

Yes

Are all the source data underlying the results available to ensure full reproducibility?

Yes

Is the study design appropriate and is the work technically sound?

Partly

Are the conclusions drawn adequately supported by the results?

Partly

Are sufficient details of methods and analysis provided to allow replication by others?

Yes

Reviewer Expertise:

Carbon and nitrogen cycles in terrestrial ecosystems

I confirm that I have read this submission and believe that I have an appropriate level of expertise to confirm that it is of an acceptable scientific standard.

# Peer review: Reviewer response for version 2

**DOI:** 10.5256/f1000research.29978.r72581

**Published:** 2020-10-14

**Authors:** Katalin Juhos

**Affiliations:** 1 Department of Agro-Environmental Studies, Szent István University, Budapest, Hungary

I have no further comments to make.

Is the work clearly and accurately presented and does it cite the current literature?

Partly

If applicable, is the statistical analysis and its interpretation appropriate?

Partly

Are all the source data underlying the results available to ensure full reproducibility?

Partly

Is the study design appropriate and is the work technically sound?

Partly

Are the conclusions drawn adequately supported by the results?

Partly

Are sufficient details of methods and analysis provided to allow replication by others?

Partly

Reviewer Expertise:

soil quality, nutrient management

I confirm that I have read this submission and believe that I have an appropriate level of expertise to confirm that it is of an acceptable scientific standard.

# Peer review: Reviewer response for version 2

**DOI:** 10.5256/f1000research.29978.r72582

**Published:** 2020-10-07

**Authors:** Paul S. Agachi

**Affiliations:** 1 Department of Chemical, Materials and Metallurgical Engineering, Faculty of Engineering and Technology, Botswana International University of Science and Technology, Palapye, Botswana

Agree with the changes, approve for indexing.

Is the work clearly and accurately presented and does it cite the current literature?

Yes

If applicable, is the statistical analysis and its interpretation appropriate?

Partly

Are all the source data underlying the results available to ensure full reproducibility?

Yes

Is the study design appropriate and is the work technically sound?

Yes

Are the conclusions drawn adequately supported by the results?

Yes

Are sufficient details of methods and analysis provided to allow replication by others?

Partly

Reviewer Expertise:

Chemical engineering, clean coal technology, process control, modeling, optimization.

I confirm that I have read this submission and believe that I have an appropriate level of expertise to confirm that it is of an acceptable scientific standard.

# Peer review: Reviewer response for version 1

**DOI:** 10.5256/f1000research.27875.r71958

**Published:** 2020-09-29

**Authors:** Paul S. Agachi

**Affiliations:** 1 Department of Chemical, Materials and Metallurgical Engineering, Faculty of Engineering and Technology, Botswana International University of Science and Technology, Palapye, Botswana

The paper deals with the assessment of the appropriation of the sandy Pallag soil for raising crops, due to the humus and Nitrogen content. The ratio of nitrate-N and nitrite-N recommends the soil for crops. 

 The importance of this study is given mainly by the fact it is done on experimental plots, in real conditions.

 My objections are related to the transparency of the Equations 1 and 2. Additional explanations should be added. Why the authors calculated this way the organic and the humus %? The values of the constants should be explained. Maybe they are known for the category of the soil scientists, but for larger dissemination and understanding, additional explanations are needed.

Is the work clearly and accurately presented and does it cite the current literature?

Yes

If applicable, is the statistical analysis and its interpretation appropriate?

Partly

Are all the source data underlying the results available to ensure full reproducibility?

Yes

Is the study design appropriate and is the work technically sound?

Yes

Are the conclusions drawn adequately supported by the results?

Yes

Are sufficient details of methods and analysis provided to allow replication by others?

Partly

Reviewer Expertise:

Chemical engineering, clean coal technology, process control, modeling, optimization.

I confirm that I have read this submission and believe that I have an appropriate level of expertise to confirm that it is of an acceptable scientific standard, however I have significant reservations, as outlined above.

# Peer review: Reviewer response for version 1

**DOI:** 10.5256/f1000research.27875.r70069

**Published:** 2020-09-18

**Authors:** Yuhua Kong

**Affiliations:** 1 College of Forestry, Henan Agricultural University, Zhengzhou, China

- The purpose and significance of the study are not very clear and lack of novelty.    
- The basic information of sampling site is not mentioned, like land-use type, the mean annual precipitation and air temperature, etc. 
- How many replicates were carried out in the study? In table 1, the standard deviation should be included. 
- It is suggested that the Pearson correlation analysis between soil humus and all nitrogen forms be shown in the results part.  

Is the work clearly and accurately presented and does it cite the current literature?

Partly

If applicable, is the statistical analysis and its interpretation appropriate?

Yes

Are all the source data underlying the results available to ensure full reproducibility?

Yes

Is the study design appropriate and is the work technically sound?

Partly

Are the conclusions drawn adequately supported by the results?

Partly

Are sufficient details of methods and analysis provided to allow replication by others?

Yes

Reviewer Expertise:

Carbon and nitrogen cycles in terrestrial ecosystems

I confirm that I have read this submission and believe that I have an appropriate level of expertise to confirm that it is of an acceptable scientific standard, however I have significant reservations, as outlined above.

# Peer review: Reviewer response for version 1

**DOI:** 10.5256/f1000research.27875.r69180

**Published:** 2020-08-24

**Authors:** Katalin Juhos

**Affiliations:** 1 Department of Agro-Environmental Studies, Szent István University, Budapest, Hungary

The purpose of the manuscript is not entirely clear:

- Exactly what land-use type the samples come from (what do control and greenhouse mean? I don't see the comparison in the results section.)
- What do you mean by "pollutant nitrogen" (= nitrite?)?
- On the basis of the soluble N-forms measured once in the upper 20 cm layer, how can it be concluded that the test soil is suitable for use? Soluble N-forms change very rapidly in soil.

N-test methods are up-to-date, but it is not clear what soil-liquid ratio was used for CaCl _2_ and KCl extracts? Nor is it clear how many soil samples were tested? 

 The detailed determination of soluble N-forms is very important. It would have been interesting to see this in the deeper layers of the soil as well. Furthermore, based on the total (Kjeldahl) N content of the soil, a picture of the source of soluble components could be obtained. Did the total soluble N content not correlate with SOM?

 "Soil organic matter (SOM)" is proposed instead of "Humus" and "soil organic carbon (SOC)" is proposed instead of "C%".

 In the description of the SOM method, K2SO4 is erroneously used instead of K-dichromate.

 When giving the averages, the number of items and the standard deviation must also be included (Table 1).

Is the work clearly and accurately presented and does it cite the current literature?

Partly

If applicable, is the statistical analysis and its interpretation appropriate?

Partly

Are all the source data underlying the results available to ensure full reproducibility?

Partly

Is the study design appropriate and is the work technically sound?

Partly

Are the conclusions drawn adequately supported by the results?

Partly

Are sufficient details of methods and analysis provided to allow replication by others?

Partly

Reviewer Expertise:

soil quality, nutrient management

I confirm that I have read this submission and believe that I have an appropriate level of expertise to confirm that it is of an acceptable scientific standard, however I have significant reservations, as outlined above.
